# Evaluation of Methods for the Extraction and Purification of DNA from the Human Microbiome

**DOI:** 10.1371/journal.pone.0033865

**Published:** 2012-03-23

**Authors:** Sanqing Yuan, Dora B. Cohen, Jacques Ravel, Zaid Abdo, Larry J. Forney

**Affiliations:** 1 Department of Biological Sciences, University of Idaho, Moscow, Idaho, United States of America; 2 Department of Mathematics, University of Idaho, Moscow, Idaho, United States of America; 3 Department of Statistics, University of Idaho, Moscow, Idaho, United States of America; 4 Institute for Bioinformatics and Evolutionary Studies, University of Idaho, Moscow, Idaho, United States of America; 5 Institute for Genome Sciences, University of Maryland School of Medicine, Baltimore, Maryland, United States of America; Argonne National Laboratory, United States of America

## Abstract

**Background:**

DNA extraction is an essential step in all cultivation-independent approaches to characterize microbial diversity, including that associated with the human body. A fundamental challenge in using these approaches has been to isolate DNA that is representative of the microbial community sampled.

**Methodology/Principal Findings:**

In this study, we statistically evaluated six commonly used DNA extraction procedures using eleven human-associated bacterial species and a mock community that contained equal numbers of those eleven species. These methods were compared on the basis of DNA yield, DNA shearing, reproducibility, and most importantly representation of microbial diversity. The analysis of 16S rRNA gene sequences from a mock community showed that the observed species abundances were significantly different from the expected species abundances for all six DNA extraction methods used.

**Conclusions/Significance:**

Protocols that included bead beating and/or mutanolysin produced significantly better bacterial community structure representation than methods without both of them. The reproducibility of all six methods was similar, and results from different experimenters and different times were in good agreement. Based on the evaluations done it appears that DNA extraction procedures for bacterial community analysis of human associated samples should include bead beating and/or mutanolysin to effectively lyse cells.

## Introduction

The microorganisms that colonize various anatomical sites of the human body play important roles in human health and disease [1]. For example, bacteria in the human intestine contribute to digestion of inaccessible compounds [2] and development of the host immune system [3], [4], while vaginal microbiota helps prevent urogenital diseases and maintain health in women [5], [6], [7]. In recent years there has been increasing interest in knowing more about how differences between individuals, or within individuals over time influence the maintenance of health and risk to disease. Such studies require a detailed understanding of the microbial diversity found at various anatomically distinct sites of the human body. The cultivation-dependent methods commonly used in clinical and research laboratories have provided a valuable but incomplete picture of the vast diversity found in the human microbiome because many, if not most human-associated microorganisms have not yet been successfully cultured in the laboratory [8], [9], [10], [11]. These methods are also limited because most do not lend themselves to the analysis of large numbers of samples because they are labor-intensive and costly. However, the application of cultivation-independent molecular approaches based on the phylogenetic analysis of the 16S rRNA gene sequences provides a way to access the uncultured majority [12], [13], allowing for more comprehensive comparative studies of microbial communities associated with the human body [14], [15], [16].

Various cultivation-independent approaches to characterizing diversity in microbial communities all require extraction of genomic DNA from the samples of interest. Previous studies have shown that differences in the structures of bacterial cell walls cause bacterial cell lysis to be more or less efficient [17], [18], [19]. This can distort the apparent composition of microbial communities [17], [20], [21], [22], [23], [24] and introduce bias in estimates of relative abundances of microbes in samples [17], [19], [25]. However, despite the critical nature of this first step, the selection of a suitable procedure for the extraction of DNA from human samples has not received enough attention [18], [26]. Indeed, in many previous investigations of the human microbiome, the genomic DNA extraction methods used were chosen without an obvious rationale, and used without validation.

Multiple criteria, including DNA yield, DNA shearing, reproducibility, and representativeness can be used to evaluate DNA extraction methods. Numerous investigators have tried to increase the DNA yield through use of physical disruption methods such as bead beating and sonication to improve the lysis of bacterial cells. However, such treatments can shear genomic DNA into small fragments and this may lead to the formation of chimeric products during PCR amplification of gene targets [27], [28]. In addition, it is important to assess the variation between analysts and over time. This is especially important when trying to track differences across sampling sites, time scales or treatments, and to compare results obtained by different laboratories. But achieving an accurate representation of bacterial profiles is arguably the most critical criterion [29], [30], because ultimately the objective is to obtain DNA that fairly represents the microbial diversity in samples with the least bias for composition and abundance. Unfortunately, most studies have evaluated the efficacy of different DNA extraction methods using environmental samples comprised of unknown microbes [17], [31], [32], which make evaluation of representativeness impossible.

In this study, we created a mock community that contained equal numbers of cells of eleven human-associated bacterial species. Six commonly used DNA extraction methods that employed different mechanisms for cell lysis and DNA purification were statistically evaluated according to the following criteria: DNA yield, DNA shearing, representation of microbial diversity, and reproducibility. The objective of this study was to identify DNA extraction methods suitable for comparative analysis of human microbiome samples.

## Results

### DNA yield

We compared six different DNA isolation methods commonly used to extract bacterial total DNA from human samples (Table 1). The yield of genomic DNA from 11 microbial species (Table 2) representing different human body sites and a mixture of these were determined. Since the volumes of all DNA samples were standardized, we used DNA concentrations to compare yields. Analysis of variance (ANOVA) showed that the DNA yield varied significantly depending on the DNA extraction method used (p = 0.0017). To explore this in more detail, Tukey's HSD procedure was used to perform pair-wise comparisons between the six methods with respect to DNA recovered from each species. As shown in Table 3, the phenol-chloroform-isoamyl alcohol extraction method (method 4) produced the highest DNA concentrations on average from all but one (*Atopobium vaginae* BAA-55) of the twelve samples. For seven of the 11 bacterial species, DNA yields obtained using method 4 were significantly higher than DNA yields obtained using the other five methods that employed commercial kits. For example, DNA yield using the phenol-chloroform-isoamyl alcohol extraction method was at least 5.7, 5.4 and 3.3-fold higher on average for *S. aureus* ATCC 12600, *Pr. acnes* ATCC 6919 and *C. tuberculostearicum* ATCC 35692, respectively. Among the five methods based on commercial kits, method 1 and 5 performed better than the other three methods for most species based on DNA yields. In comparison, the lowest DNA concentrations were achieved with method 3 for seven of the twelve samples.

**Table 1:** Features of the six DNA extraction methods used.

Method	Cell lysis^a^	DNA purification	References
1	E2, C	Silica column	[71]
2	B, E1, E2, E3, C	Silica column	[16], [60]
3	C	Silica column	[14], [62]
4	B, C	Precipitation^b^	[58], [61]
5	E1, C	Silica column	[15], [59]
6	B, E1, C	Silica column	This study

a Cell lysis method: B, bead beating; E1, lysozyme; E2, mutanolysin; E3, lysostaphin; C, chemical.

b Phenol-chloroform purification and isopropanol precipitation.

**Table 2:** Bacterial strains and cultivation condition used.

Type strains	Gram-stain	Atmosphere^a^	Medium^b^
*Escherichia coli* ATCC 47076	−	aerobic	Broth: LB
*Staphylococcus aureus* ATCC 12600	+	aerobic	Broth: TSB
*Pseudomonas aeruginosa* ATCC 10145	−	aerobic	Broth: Nutrient
*Streptococcus agalactiae* ATCC 12403	+	aerobic	Broth: BHI
*Corynebacterium tuberculostearicum* ATCC 35692	+	aerobic	Agar: BHI +5% sheep blood
*Enterococcus faecalis* ATCC 19433	+	aerobic	Broth: BHI
*Lactobacillus iners* DSMZ 13335	+	anaerobic	Broth: BHI +5% horse serum
*Lactobacillus crispatus* ATCC 33820	+	anaerobic	Broth: MRS
*Atopobium vaginae* BAA-55	+	anaerobic	Broth: TSB +5% horse serum
*Gardnerella vaginalis* ATCC 14018	+	anaerobic	Broth: ATCC NYC III medium
*Propionibacterium acnes* ATCC 6919	+	anaerobic	Broth: Reinforced Clostridial medium

a Anaerobic strains were cultivated in GasPak anaerobic chamber (Becton Dickinson, Franklin Lakes, NJ) with Pack-Anaero sachet (MGC Inc., New York, NY).

b Medium: LB, Luria-Bertani; TSB, Trypticase soy base; BHI, Brain heart infusion; MRS, De Man, Rogosa and Sharpe.

**Table 3:** Comparison of DNA yields of type strains obtained using six DNA extraction methods.

Strain	Method	DNA conc. (µg/ml)^a^	Pairwise comparison^b^	Strain	Method	DNA conc. (µg/ml)	Pairwise comparison
*Es. coli* ATCC 47076	4	4.26	A	*L. iners* DSMZ 13335	4	6.45	A
	5	3.47	A		5	3.65	A B
	1	2.96	A		1	3.32	B
	6	1.46	B		2	1.28	C
	3	0.88	C		3	1.23	C
	2	0.81	C		6	1.11	C
*Sta. aureus* ATCC 12600	4	4.81	A	*L. crispatus* ATCC 33820	4	3.65	A
	1	0.85	B		1	2.22	B
	2	0.84	B		2	1.24	C
	5	0.4	C		5	1.16	C
	6	0.29	C		3	0.27	D
	3	0.19	D		6	0.23	D
*Ps. aeruginosa* ATCC 10145	4	5.39	A	*A. vaginae* BAA-55	1	1.66	A
	1	2.38	B		4	1.01	B C
	5	2.07	B		5	0.73	C D
	6	1.24	C		3	0.51	D E
	3	0.86	C D		2	0.38	E
	2	0.71	D		6	0.18	F
*Str. agalactiae* ATCC 12403	4	5.91	A	*G. vaginalis* ATCC 14018	4	1.7	A
	1	2.5	A		1	1.65	A
	2	0.75	B		5	0.62	B
	5	0.46	B		2	0.58	B
	6	0.34	B		6	0.1	C
	3	0.05	C		3	0.09	C
*C. tuberculostearicum* ATCC 35692	4	4.54	A	*Pr. acnes* ATCC 6919	4	2.2	A
	1	1.38	B		5	0.41	B
	5	0.79	C		2	0.36	B
	2	0.77	C		1	0.35	BC
	6	0.69	C		6	0.21	CD
	3	0.19	D		3	0.14	D
*En. faecalis* ATCC 19433	4	8.33	A	Mock community	4	2.77	A
	1	3.12	B C		1	1.52	B
	5	1.97	C D		5	1.04	B
	2	1.71	D E		2	0.6	C
	6	1.09	E F		6	0.38	D
	3	0.97	F		3	0.37	D

a DNA concentrations are means calculated using data from eight replicates.

b Means with the same letter are not significantly different.

### DNA shearing

The degree of genomic DNA shearing during the various extraction procedures was assessed by electrophoresis using a 0.8% (wt/vol) agarose gel and compared to λ-*Hind* III DNA size standards (data not shown). The maximum size of genomic DNA in all cases was between 9.4 kb and 23 kb. DNA shearing occurred in all extractions and DNA fragments were as short as 125 bp. Higher molecular weight genomic DNA was observed from *S. aureus* ATCC 12600, *S. agalactiae* ATCC 12403 and *C. tuberculostearicum* ATCC 35692 using methods 1, 5 and 6. In contrast, the genomic DNA of *L. iners* DSMZ 13335, *L. crispatus* ATCC 33820, *A. vaginae* BAA-55 and *G. vaginalis* ATCC 14018 demonstrated more shearing when methods 1, 4, 5 and 6 were used.

### Representation of microbial diversity

To evaluate how well each method yielded DNA that was representative of that in a mixture of organisms, we created a mock community in such a way that expected abundances could be calculated. Since we included an equal number of cells of each species in the mock community, a simple prediction should be that the expected relative abundance of the 16S rRNA gene per strain would be directly proportional to their copy number of 16S rRNA genes. Using this approach the expected relative abundances were calculated and are shown in Table 4. By counting the number of reads of 16S rRNA genes from each species and normalizing by the total number of reads per sample, we could estimate the observed relative abundances of 16S rRNA gene reads for each species in the mock community (Table 4). Using a likelihood ratio test with bootstrapping, and accounting for overdispersion in sampling (see Appendix S2), we tested whether observed abundances matched expected abundances. For all DNA extraction methods the observed abundances distribution was significantly different from expected abundances (all p-values≪0.01).

**Table 4:** 16S rRNA gene copy numbers, expected and observed proportions of 16S rRNA gene sequence reads for each type strain.

Type strains	16S rRNA gene copy number a	Expected proportion (%)	Observed proportions (%)^b^					
			Method 1	Method 2	Method 3	Method 4	Method 5	Method 6
*Escherichia coli* ATCC 47076	7	13.5	9.3 (±2.3)	8.6 (±2.0)	9.5 (±1.4)	6.2 (±1.6)	3.7 (±0.9)	5.8 (±1.9)
*Staphylococcus aureus* ATCC 12600	6	11.5	7.5 (±1.0)	11.4 (±1.2)	5.0 (±1.1)	11.4 (±2.6)	3.4 (±0.9)	4.6 (±1.2)
*Pseudomonas aeruginosa* ATCC 10145	4	7.7	2.3 (±0.9)	2.7 (±0.9)	2.9 (±1.0)	1.2 (±0.7)	0.9 (±0.2)	2.4 (±0.9)
*Streptococcus agalactiae* ATCC 12403	7	13.5	15.7(±3.1)	14.8 (±1.4)	1.6 (±1.2)	19.4 (±2.8)	7.1 (±4.0)	15.7 (±3.3)
*Corynebacterium tuberculostearicum* ATCC 35692	8	15.4	9.4 (±1.6)	9.6 (±2.2)	5.3 (±1.2)	13.8 (±1.8)	9.6 (±2.0)	15.3 (±2.5)
*Enterococcus faecalis* ATCC 19433	4	7.7	6.1 (±1.5)	5.9 (±1.6)	10.4 (±3.2)	7.6 (±2.5)	6.3 (±1.5)	10.2 (±2.1)
*Lactobacillus iners* DSMZ 13335	5	9.6	21.5 (±4.1)	18.9 (±3.7)	45.8 (±2.3)	27.6 (±6.4)	48.0 (±9.2)	37.7 (±6.3)
*Lactobacillus crispatus* ATCC 33820	4	7.7	5.2 (±2.3)	7.0 (±2.6)	2.5 (±0.9)	4.0 (±1.3)	9.0 (±3.8)	3.6 (±1.5)
*Atopobium vaginae* BAA-55	2	3.8	10.8 (±2.5)	8.3 (±1.7)	14.6 (±2.0)	2.7 (±1.0)	5.1 (±1.1)	1.9 (±1.2)
*Gardnerella vaginalis* ATCC 14018	2	3.8	11.9 (±1.6)	11.6 (±0.9)	1.8 (±0.7)	3.4 (±1.4)	5.6 (±1.6)	0.5 (±0.3)
*Propionibacterium acnes* ATCC 6919	3	5.8	0.3 (±0.3)	1.2 (±0.8)	0.7 (±0.3)	2.7 (±0.8)	1.2 (±0.5)	2.3 (±1.6)

a If more than one possible copy numbers are available for one species in *rrn* database (http://ribosome.mmg.msu.edu/rrndb/index.php), the larger one was chosen.

b Average proportions and standard deviations are calculated based on eight replicates.

**Figure 1:**
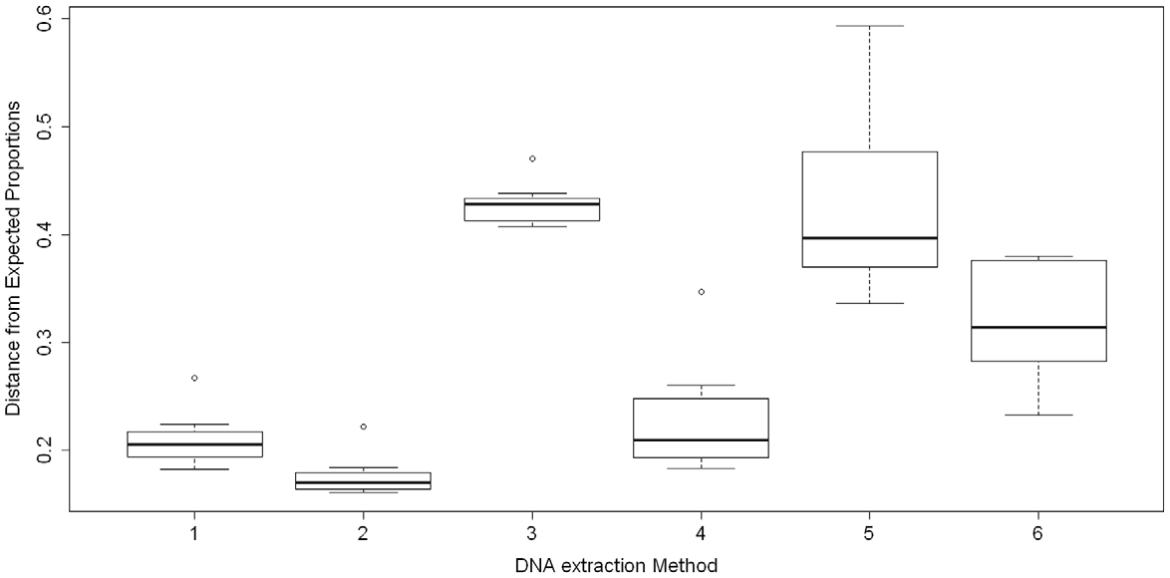
Boxplot of Euclidean distances between observed and expected species proportions. Euclidean distances between observed and expected proportions were calculated for each of eight replicates of each method.

Furthermore, to evaluate whether some DNA extraction methods better represented bacterial community structure than other DNA extraction methods, we calculated Euclidean distances between observed and expected proportions for all 48 samples (8 replicates per method). Based on a boxplot of Euclidean distances (Figure 1) and pair-wise comparisons of Euclidean distances using Wilcoxon rank sum test, we found that method 1 and method 2 produced a significantly better representation of bacterial community structure than method 3, method 5 and method 6 (all p-values<0.01). Method 4 was better than methods 5 and 3 (p-value<0.03), but not method 6 (p-value = 0.1049).

Curiously, *L. iners* DSMZ 13335 was significantly over-represented in all samples relative to the expectation. For example, the relative abundances of *L. iners* DSMZ 13335 generated from Method 3 and Method 5 were at least 4.7-fold higher than its expected relative abundance. This can not be explained. In contrast, *C. tuberculostearicum* ATCC 35692, *E.coli* ATCC 47076, *P. aeruginosa* ATCC 10145 and *P. acnes* ATCC 6919 were under-represented in all samples.

### Reproducibility

**Figure 2:**
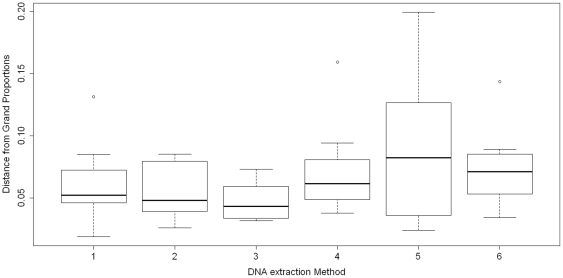
Boxplot of Euclidean distances between observed and grand proportions. To calculate grand proportions, the total counts of 16S rRNA gene reads of each species were calculated for eight replicates of each method. Then grand proportions were calculated based on total counts of 16S rRNA gene reads of each species per method. Grand proportions were used to calculate Euclidean distances between observed and grand proportions.

To evaluate the reproducibility of the DNA extraction methods we performed eight replicated DNA extractions from samples of the mock community for each DNA extraction method, and these were performed by two experimenters on two different days. Pair-wise comparison of variances showed no significant differences between any two of the six DNA extraction methods based on an F-K test (all p-values≫0.00067). However, the results obtained using method 5 had the largest variance (Figure 2). Analyses of the data using the Wilcoxon rank sum test showed there was usually good agreement between results from different experimenters and for extractions done on different days (all p-values>0.05). The one exception was the poor agreement between results from different experimenters using method 4 (p-value = 0.0286).

### Correlation between DNA yields and representation of microbial diversity

DNA yield is often used as a criterion to assess the effectiveness of procedures for the isolation of genomic DNA from microbial communities. To determine if higher DNA yield ensured better representation of microbial diversity, we calculated Spearman's rank correlation coefficients to compare DNA yield and representativeness. Euclidean distances between observed proportions and expected proportions were used to represent microbial diversity. The correlations were calculated within a method or between different methods. There was no significant correlation between DNA yields and distances within (all p-values>0.1) or between DNA extraction methods (p-value = 0.3556).

### Comparison of cell lysis efficiency of different lytic modes

**Figure 3:**
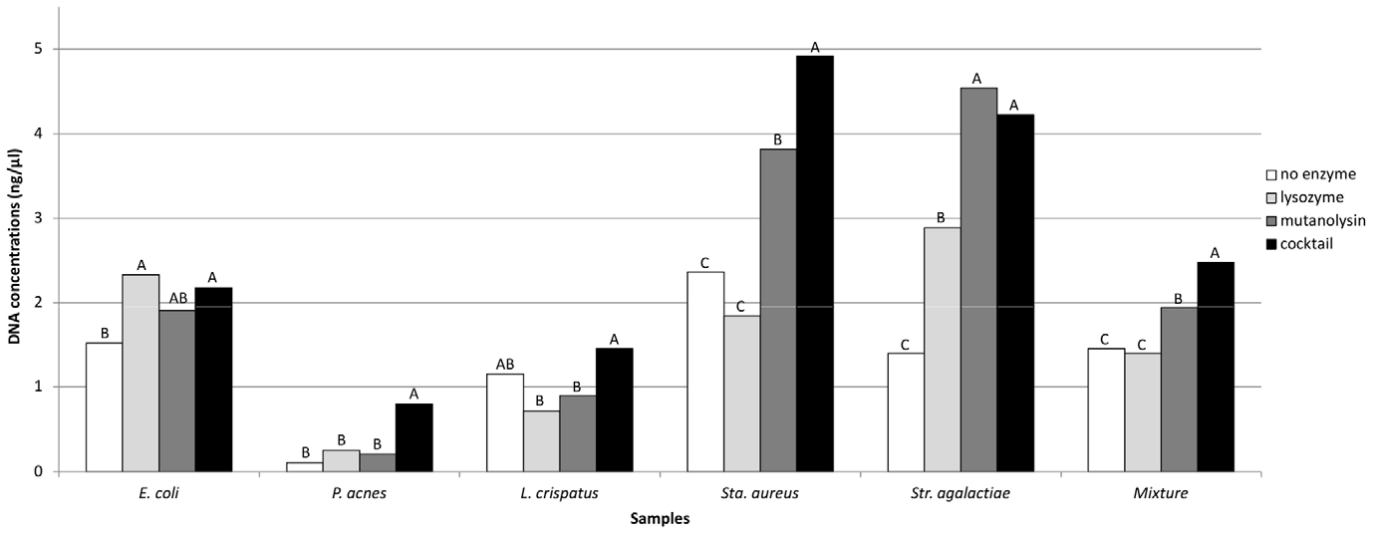
DNA extractions using different enzymatic lysis modes. The mean concentrations (columns) were calculated based on nine replicated extractions per sample per mode. Pair-wise comparisons of DNA concentrations between modes per sample were performed by using Wilcoxon rank sum test. Bonferroni correction was used for multiple testing. Letters at the top of columns indicate whether there is significantly difference between columns per sample. Means with the same letter are not significantly different.

To investigate the lysis efficiency of different lytic modes in more details, four different enzymatic lysis modes, including no lytic enzyme, lysozyme alone, mutanolysin alone and a cocktail of lysozyme, mutanolysin and lysostaphin, were evaluated using a double blind experimental design (see Appendix S1). Consistent results were obtained by different experimenters at different times using each of the four enzymatic lysis modes (Figure S1). However, DNA extractions done using a cocktail of lytic enzymes consistently lysed cells of different species more effectively (Figure 3).

## Discussion

Numerous studies have been done to evaluate microbial DNA extraction methods using various kinds of samples [17], [21], [22], [23], [24], [33], [34], [35], [36], [37]. The criteria employed in these studies included DNA yield [17], [21], [23], [24], [33], [34], [35], [36], [37], DNA purity [23], [24], [33], [36], [37], cell lysis efficiency [17], [35], [38], reproducibility [17], [21], [22], [24], [37] and species richness [17], [21], [23], [24], [37]. However, the representation of microbial diversity, which is often the main goal of community analysis, is generally not considered as a criterion for evaluation of DNA extraction methods. This is mainly due to the use of environmental samples for the assessment of protocols, and such samples include unknown numbers and kinds of indigenous microbes. Without a control community with known species composition and abundances, it is impossible to evaluate the ability of different DNA extraction methods to fairly represent the microbial diversity in a sample.

Here we sought to compare the ability of six DNA extraction methods previously used in studies of the human microbiome and environmental samples to recover DNA from known organisms and yield genomic DNA representative of mock community. We found that observed species abundances from all six DNA extraction methods did not match the expected species abundances, and the differences between them were significant. This bias could be ascribed to many factors in addition to DNA extraction efficiency. For example, the copy number of the chromosome can vary depending on growth phase [39], [40], and bias can occur during PCR amplification since the “universal” primers used are not really universal [28]. In addition, genome size and *rrn* gene copy number also have an effect on PCR [41]. Because this study was not designed to evaluate the effect of those factors mentioned above on observed relative abundance, we tried to minimize biases introduced by those factors. First, the cells used were harvested in post-exponential phase of growth to reduce the variation of chromosome copy number. Second, a mixture of forward primers (27F) were used to minimize the PCR amplification bias [42]. Third, information on the *rrn* gene copy number of each strain was taken into account to calculate the expected relative abundances. Therefore, in this case, DNA extraction efficiency was likely to be the main factor that introduced bias between observed and expected relative abundance.

Previous studies have shown that observed microbial composition is mainly affected by the efficiency of cell lysis instead of DNA recovery [21], [23], [34], [43]. Generally, gram-positive bacteria are expected to be under-represented in the observed relative abundance data because they are more recalcitrant to lysis while gram-negative bacteria should be over-represented. However, this was not always the case in our study. For example, gram-positive *L. iners* DSMZ 13335 was over-represented (2.2–4.8 fold) relative to its expected relative abundance in all samples. This may be partly explained by the gram-variable property of *L. iners* reported before [44]. In contrast, two gram-negative bacteria (*E. coli* ATCC 47076 and *P. aeruginosa* ATCC 10145) were markedly under-represented in all samples. Similar results were reported by Morgan *et al.* [20]. The reasons for these results are unknown.

We found that extraction methods that included bead beating and/or mutanolysin (methods 1, 2, and 4) produced significantly better representations of bacterial community structure than methods without both of these steps (methods 3 and 5; Figure 1). Method 2, which included bead beating and a cocktail of lytic enzymes (mutanolysin plus lysozyme and lysostaphin), gave the best representation of microbial diversity compared to the other five methods. Previous studies have reported that higher DNA extraction efficiencies can be achieved if the procedure used includes a step for the mechanical disruption of microbial cells by bead beating [22], [34], [36]. This was especially true for the efficient extraction of DNA from gram-positive bacteria that typically have cell walls with thick layers of peptidoglycan. This higher lysis efficiency provides a more comprehensive and even profile of the microbial diversity [21], [22], [36]. In method 6, although bead beating and enzymatic lysis were included, the beads used in this method were much larger than the beads used in method 2 and lysozyme alone was used for enzymatic lysis. This may partly explain why method 6 produced a significantly worse representation of bacterial community structure compared to methods 1 and 2. Figure 3 showed that cell lysis is not very efficient when lysozyme is used alone, especially for gram-positive bacterial cells. However, a cocktail of lytic enzymes demonstrated consistently good cell lysis efficiency for all samples. This probably reflects differences in the structure of peptidoglycan between different bacterial species, which results in more or less recalcitrance to lysozyme. It is well known that c-type lysozyme such as hen egg-white lysozyme is a 1,4-β-N-acetylmuramidase, cleaving the glycosidic bond between the C-1 of N-acetylmuramic acid and the C-4 of N-acetylglucosamine in the bacterial peptidoglycan [45]. However, some bacteria have a modified peptidoglycan structure that is not sensitive to c-type lysozyme [46], [47]. For example, many bacteria are known to have O-acetylated peptidoglycan; including some important human-associated bacteria such as *Neisseria gonorrhoeae*, *Proteus mirabilis* and *S. aureus* [46] These bacteria are sensitive to mutanolysin rather than lysozyme [48]. Mutanolysin also has lytic activity against some species of *Streptococcus* and *Lactobacillus* [49], which can be commonly found in the human gut and vagina [14], [50]. Lysostaphin is a glycylglycine endopeptidase that is able to specifically cleave the cross-linking pentaglycine bridges in the cell wall of *staphylococci* [51], [52]. Using a cocktail of lytic enzymes is likely to reduce insufficient or preferential cell lysis and lead to a better representation of bacterial diversity.

We found no correlation between DNA yields and the representation of microbial diversity when within (all p-values>0.1) or between method (p-value = 0.3356) comparisons were made. In addition, the species proportions observed with all six methods were more consistent than DNA yields from replicate extractions. This is consistent with findings of other studies in which investigators have shown there are no correlations between DNA yields and observed species richness [21], [23], [33], [34], [43], [53], [54]. These results suggest one cannot be assured that microbial diversity will be better represented simply because the DNA yield from a given procedure is greater. For example, it has been reported many times that DNA extraction methods using phenol-chloroform purification and ethanol precipitation harvested relatively more bacterial DNA than DNA extraction methods using silica columns for DNA recovery, however, higher DNA yields did not provided higher observed species richness in these studies [21], [23], [33], [36], [38], [55]. To the contrary in this study we found that methods that gave lower DNA yields actually more fairly represented the microbial diversity in a mock community. For example, method 2 performed best even though the DNA yield from the mock community was relatively low.

In sum, protocols that employed bead beating and/or mutanolysin for cell lysis better represented bacterial community structure than methods without both of them. On this basis, methods 1 and method 2 can be recommended for studies done to characterize microbial diversity using cultivation independent methods. That said, it should be noted that no method tested in this study provided an accurate representation of the bacterial diversity present in the mock community used. This result indicates that investigators should use caution in drawing conclusions about the relative abundances of bacterial populations in communities. Fortunately, the reproducibility of all the methods when used by different experimenters on different days suggests that comparative analyses between samples and over time can be done with a reasonable degree of confidence.

## Materials and Methods

### Strains and cultivation conditions

Eleven type strains (Table 2) chosen in this study are represent microbial species commonly found at different human body sites, including the gut [14], [56], [57], [58], skin [15] and vagina [16], [59], [60], [61], [62]. Two of them are gram-negative and the others are gram-positive, so two different kinds of cell wall architecture were represented. The cultivation conditions used are shown in Table 2. The cultivation temperature for all type strains was 37°C.

### Cell counting and preparation of the mock community

The cells of type strains that readily cultivated in liquid medium (Table 2) were collected by centrifugation and then re-suspended in phosphate buffered saline (PBS) on ice. The cells of *C. tuberculostearicum* ATCC 35692 were collected from plates and re-suspended in PBS on ice. The cell density of each type strain was determined by using a bright-line counting chamber (Hausser Scientific, Horsham, PA). We adjusted the cell density of each type strain to 10^8^ cells ml^−1^ by diluting with PBS. In addition, a mock community was prepared by mixing equal volumes of cell suspensions of all eleven type strains, resulting in an equal number of cells of each type strain in the mixture. Aliquots (0.5 ml) of these cell suspensions were placed in microcentrifuge tubes and frozen at −80°C.

### DNA extraction methods

Six DNA extraction methods (Table 1) were compared in this study, representing different kinds and combinations of cell lysis mechanisms and DNA purification methods commonly used in the published literature on the human microbiome. Each method was evaluated using all 11 type strains and a mock community sample. The isolated genomic DNA was in a final volume of 200 µl.

**Method 1.** The QIAamp DNA mini kit (Qiagen, Valencia, CA) was used in this method with minor modifications. Briefly, 6 µl mutanolysin (25 KU/ml, Sigma-Aldrich) was added to a 500 µl aliquot of cells and the mixture was incubated for 30 min at 37°C. After this, 50 µl Proteinase K (20 mg/ml) and 500 µl AL buffer (Qiagen, Valencia, CA) were added and the sample was incubated for 30 min at 56°C. Then, 500 µl of ethanol was added and DNA was purified by using the columns provided in the kit (Qiagen, Valencia, CA) according to the manufacturer's instructions.

**Method 2.** A two-step cell lysis procedure was employed before use of the QIAamp DNA mini kit (Qiagen, Valencia, CA). First, 50 µl lysozyme (10 mg/ml, Sigma-Aldrich), 6 µl mutanolysin (25 KU/ml, Sigma-Aldrich), and 3 µl lysostaphin (4000 U/ml, Sigma-Aldrich) were added to a 500 µl aliquot of cell suspension followed by incubation for 1 hour at 37°C. Second, 600 mg of 0.1-mm-diameter zirconia/silica beads (BioSpec, Bartlesville, OK) were added to the lysate and the microbial cells were mechanically disrupted using Mini-BeadBeater-96 (BioSpec, Bartlesville, OK) at 2100 rpm for 1 minute. Further isolation and purification of the total genomic DNA from lysates was done using QIAamp DNA mini kits (Qiagen, Valencia, CA).

**Method 3.** Genomic DNA was extracted by using the QIAamp DNA stool kit (Qiagen, Valencia, CA) with a 95°C lysis step according to the manufacturer's instructions. Briefly, 500 µl ASL buffer was add to a 500 µl aliquot of cells suspension and the mixture was heated for 5 min at 95°C. Then, 100 µl Proteinase K (20 mg/ml) and 1 ml AL buffer were added and the mixture was incubated for 10 min at 70°C. After this, 1 ml of ethanol was added and the rest of the isolation protocol was continued as described by the manufacturer.

**Method 4.** A 210 µl aliquot of 20% SDS, 500 µl of a mixture of phenol∶ chloroform∶ isoamyl alcohol (25∶24∶1)], and 600 mg of 0.1-mm-diameter zirconia/silica beads (BioSpec, Bartlesville, OK) were add to a 500 µl aliquot of cells suspension. Microbial cells were then disrupted by using Mini-BeadBeater-96 (BioSpec, Bartlesville, OK) set on 2100 rpm for 1 min. Next, the mixture was centrifuged at full speed (14000 rpm) for 5 min to separate phases. The top aqueous layer was transferred to a clean 2 ml micro-centrifuge tube. Then, 0.1 volume of 3 M sodium acetate and an equal volume of ice-cold isopropanol were added to the mixture. After incubation at −20°C for 10 min, the mixture was centrifuged at 4°C at 14,000 rpm for 15 min to collect the DNA pellet, which was then washed with 1 ml ice-cold 70% (v/v) ethanol and air dried. Finally, DNA pellets were re-suspended in 200 µl AE buffer (Qiagen, Valencia, CA).

**Method 5.** DNA was extracted by using the DNeasy Tissue Kit (Qiagen, Valencia, CA) and the manufacturer's protocol for isolation of genomic DNA from Gram-positive bacteria was followed. Briefly, 50 µl lysozyme (10 mg/ml, Sigma-Aldrich) was added to a 500 µl aliquot of cells and the mixture was incubated for 30 min at 37°C. After the addition of 50 µl Proteinase K (20 mg/ml) and 500 µl AL buffer, the mixture was incubated for 30 min at 56°C. Then, 500 µl of ethanol was added to the lysate and the genomic DNA was purified using the columns in the kit according to the manufacturer's instructions.

**Method 6.** In this method, an enzymatic lysis was conducted before the PowerSoil™ DNA Isolation Kit (MO BIO Laboratories, Inc., Carlsbad, CA) was used. Briefly, 50 µl of lysozyme (10 mg/ml, Sigma-Aldrich) was added to a 500 µl aliquot of bacterial cells followed by incubation for 1 hour at 37°C. The remainder of the DNA extraction was continued beginning with step 2 of the manufacturer's protocol.

This DNA extraction experiment was finished in 12 days, in which only one DNA extraction method was used per day. The selection of DNA extraction methods was made by randomly assigning each of the six DNA extraction methods to two of 12 days. On a given day, two experimenters used a given method to extract DNA from two replicates of each sample. This was repeated once, so eight replicate samples were analyzed using each method.

### Determination of DNA yield and DNA fragment distribution

The quantity of genomic DNA in each preparation was estimated by using a PicoGreen dsDNA quantitation kit (Invitrogen, Carlsbad, CA). Fluorescence was measured using the Synergy™ HT Multi-Mode Microplate Reader (BioTek, Winooski, VT) at an excitation wavelength of 485 nm and emission wavelength of 528 nm. To evaluate DNA shearing the distribution of DNA fragment sizes were assessed by electrophoresis (3 V/cm for 1.5 h) of genomic DNA on a 0.8% (wt/vol) agarose gel followed by staining with ethidium bromide and visualization using UV light. The NEB λ-*Hind*III DNA size standards (New England Biolabs, Ipswich, MA) were used to estimate fragments sizes.

### 16S rRNA operon copy number determination for type strains

The 16S rRNA gene copy numbers for Escherichia coli ATCC 47076, Staphylococcus aureus ATCC 12600, Pseudomonas aeruginosa ATCC 10145, Streptoccus agalactiae ATCC 12403, Enterococcus faecalis ATCC 19433, Lactobacillus crispatus ATCC 33820, Gardnerella vaginalis ATCC 14018 and Propionibacterium acnes ATCC 6919 were obtained from the Ribosomal RNA Operon Copy Number Database ([63]; http://ribosome.mmg.msu.edu/rrndb/index.php) and the NCBI genome database (http://www.ncbi.nlm.nih.gov/sites/genome). The 16S rRNA gene copy numbers for the rest of type strains were determined via pulse-field gel electrophoresis (PFGE) as described by Williams [64].

### Pyrosequencing of 16S rRNA genes of mock communities

The 16S rRNA gene sequences amplified from the genomic DNA isolated from the mock community using different procedures (Table 1) were obtained by barcoded pyrosequencing. Two universal primers were used to amplify the V1–V2 hypervariable regions of 16S rRNA genes. The forward primer (5′-GCCTTGCCAGCCCGCTCAG *TC* **AGAGTTTGATCCTGGCTCAG**-3′) consisted of the 454 Life Sciences primer B (underlined), the broadly conserved bacterial primer 27F (bold), and a 2-base linker sequence (“TC”). The reverse primer (5′-GCCTCCCTCGCGCCATCAGNNNNNNNN*CA* **GCTGCCTCCCGTAGGAGT**-3′) included the 454 Life Sciences primer A (underlined), an 8 bp barcode, the bacterial primer 338R (bold), and a “CA” linker. For each sample the primer had a unique specific barcode. A mixture of forward primers were used to exclude the PCR amplification bias [42]. The mixture contained: 27f-CM (5′-AGAGTTTGATCMTGGCTCAG, where M is A or C), fourfold-degenerate primer 27f-YM (5′-AGAGTTTGATYMTGGCTCAG, where Y is C or T), and seven fold degenerate primer 27f-YM+3 [42]. This primer formulation was shown to better maintain the original rRNA gene ratio of *Lactobacillus spp.* to *Gardnerella spp.* in quantitative PCR assays [42]. Each PCR reactions consisted of 5.0 µl 10×PCR buffer II (Applied Biosystems, Foster City, CA), 6.0 µl MgCl_2_ (25 mM; Applied Biosystems, Foster City, CA), 2.5 µl Triton X-100 (1%), 0.4 µl deoxyribonucleoside triphosphates (25 mM), 0.25 µl each of primer 27F and 533R (20 pmol/µl each), 0.2 µl AmpliTaq DNA polymerase (5 U/µl; Applied Biosystems, Foster City, CA), and 1∼5 ng of template DNA in a total reaction volume of 50 µl. Samples were initially denatured at 95°C for 5 min, then amplified by using 30 cycles of 95°C for 30 s, 56°C for 30 s, and 72°C for 90 s. A final extension of 7 min at 72°C was added at the end of the program to ensure complete amplification of the target region. The PCR amplicons were quantified by using the PicoGreen dsDNA quantitation kit (Invitrogen, Carlsbad, CA) with TBS-380 mini fluorometer (Promega, Sunnyvale, CA), and equimolar amounts (100 ng) of the PCR amplicons were combined in a single tube. The 16S rRNA genes in the purified amplicon mixture were sequenced by 454 Genome Sequencer FLX System (Roche, Branford, CT).

Raw unclipped DNA sequence reads from the 454 were cleaned, assigned and filtered in the following manner. Raw SFF files were read directly into the R statistical programming language using the R package rSFFreader (unpublished), Roche quality clip points were identified and recorded, however full sequence reads (unclipped) were used for the identification of Roche 454 adapters, barcodes and amplicon primers sequence using Cross Match (version 1.080806, parameters: min matches = 15, min score = 14) from the phred/phrap/consed application suite. Cross Match alignment information was then read into R and processed to identify alignment quality, directionality, barcode assignment, and new read clip points. Base quality clipping was then performed using the application Lucy (version 1.20p, parameters: max average error = 0.002, max error at ends = 0.002). We then aligned the clipped reads to the SILVA bacterial sequence database-using mothur (version 1.12.1). Alignment end points were identified and used in subsequent filtering. Sequence reads were then filtered and only those that met the following criteria were analyzed further: (a) sequences were at least 100 bp in length; (b) max hamming distance of barcode = 1; (c) maximum number of matching error to forward primer sequences = 2; (d) had <2 ambiguous bases; (e) had <7 bp homopolymer run in sequence; (f) alignment to the SIILVA bacterial database was within 75 bp of the expected alignment start position as identified by the trimmed mean of all read alignment (trim = 10%); and (g) read alignment started within the first 5 bp and extended through read to within the final 5 bp. The RDP Bayesian classifier [65] was used to assign sequences to phylotypes. Reads were assigned to the first RDP level with a bootstrap score > = 50. In this study, a reference 16S rRNA gene sequences database, which contained the complete 16S rRNA gene sequences of the 11 type strains, was also used for further assignment of 16S rRNA gene sequences generated from pyrosequencing using speciateIT (http://sourceforge.net/projects/speciateit/). The percentages of phylotypes within each sample were then calculated.

### Data analysis

A split plot design [66] was used in this experiment. This design included one whole-plot factor (DNA extraction method), one split-plot factor (bacterial species), and complete randomization at both levels (whole-plot and split plot). Both of these factors were considered fixed. We controlled for expected difference between experimenters by using an experimenter as a random block. This resulted a mixed-effects, split plot experimental design. An analysis of variance was then conducted to evaluate significance of differences in the effect of isolation methods on DNA yield. DNA concentration data was log-transformed to accommodate the assumptions of normality and constant variance of model residuals required for this analysis. Additional pairwise comparisons were done to compare DNA concentrations between isolation methods for each bacterial species used. We used Tukey's HSD procedure to correct for multiple testing.

To compare the accuracy (representation), of the different methods in recovering the expected structure of the mock community we used a likelihood ratio test with bootstrapping, and accounted for overdispersion in sampling (see Appendix S2) as described by Schütte *et al.* [67]. Then we computed the Euclidean distances between the observed read proportions, per sample, resulting from each of these methods to the expected read proportions presented in Table 4. Accurate methods had distances close to zero. To evaluate whether some DNA extraction methods produced better bacterial community representation than other DNA extraction methods, we performed pair-wise comparisons of Euclidean distances using Wilcoxon rank sum test [68] as implemented in R [69] and utilizing a Bonferroni correction for multiple testing.

To evaluate and compare the reproducibility, precision, of these DNA extraction methods, we first pooled the reads for each OTU in the mock community within each sample observed per method. Using this pooled data we then computed the proportion of reads per OTU. The resulting vector of “grand” proportions per method was used as a baseline and Euclidean distances were calculated between proportions observed, per sample and per method, and this baseline. Reproducible methods were taken to be those with small deviation from the baseline. Reproducibility was compared using these deviations from baseline between methods by utilizing the F-K test, a nonparametric pairwise comparison of variance test [70] implemented in R [69]. We employed a Bonferroni correction for multiple testing in this case as well.

Furthermore, to evaluate whether different experimenters at different time points generated similar results using the same DNA extraction method, Euclidean distances (calculated above for representation) within each DNA extraction method generated from different experimenters at different days were compared. This analysis was performed using a Wilcoxon rank sum test. At last, correlations between DNA yields and Euclidean distances between observed proportions and expected proportions were calculated using Spearman's rank correlation coefficients in R [69]. Correlation coefficients that were not significant (*p*>0.001) were set to 0.

### Comparison of cell lysis efficiency of different lytic modes

The lysis efficiencies of four different enzymatic lysis modes, including no lytic enzyme, lysozyme alone, mutanolysin alone and a cocktail of lysozyme, mutanolysin and lysostaphin, were evaluated on the basis of DNA yield using five bacterial species and a mock community as described in Appendix S1.

## Supporting Information

Appendix S1 **Methods for comparison of cell lysis treatments.**
(DOC)

Appendix S2 **A Poisson-Binomial mixture model to account for overdispersion in microbiome sampling.**
(DOCX)

Figure S1 **Combinations of species and enzymatic lysis methods.**
(TIF)
