# Supplementary material for: Evaluation of Methods for the Extraction and Purification of DNA from the Human Microbiome
Source: PLoS One. 2012 Mar 23;7(3):e33865. doi: 10.1371/journal.pone.0033865 (PMC3311548; doi:10.1371/journal.pone.0033865)
Supplement: Appendix S1 — Methods for comparison of cell lysis treatments. (DOC) [file pone.0033865.s001.doc]

# Appendix S1

## Methods for comparison of cell lysis treatments

Five bacterial species representative of ones commonly found in different human body siteswere used. *Escherichia coli* ATCC 47076, *Staphylococcus aureus* ATCC 12600, and *Streptococcus agalactiae* ATCC 12403 were cultivated in Luria-Bertani (LB) broth, Typticase soy base (TSB) broth, and Brain heart infusion (BHI) broth, respectively, under aerobic conditions. *Lactobacillus crispatus* ATCC 33820 and *Propionibacterium acnes* ATCC 6919 were grown in de Man, Rogosa and Sharpe (MRS) broth and Reinforced Clostridial broth, respectively in GasPak anaerobic chambers (Becton Dickinson, Franklin Lakes, NJ) with Pack-Anaero sachets (MGC Inc., New York, NY). The cultivation temperature for all type strains was 37°C. The cells of type strains were collected by centrifugation and re-suspended in phosphate buffered saline (PBS) four times to remove extracellular enzymes. The optical density (OD) of each cell suspension was determined using a spectrophotometer (Biochrom, Cambridge, England) and adjusted to 0.2 OD units by diluting with PBS. Then a simple mock community was prepared by mixing equal volumes of cell suspensions of all five type strains. Aliquots (0.5 ml) of these cell suspensions and a negative control (PBS) were placed in microcentrifuge tubes and frozen at -80°C, generating seven different sample types (five type strains, one mock community and one negative control).

Four different kinds of lysis solutions were compared including no enzyme, lysozyme (5 mg/ml, Sigma-Aldrich), mutanolysin (1500U/ml, Sigma-Aldrich) and a cocktail of lytic enzymes (5 mg/ml lysozyme, 1500U/ml mutanolysin and 120U/ml lysostaphin (Sigma-Aldrich)). To extract DNA from the samples, 100 µl one of the four enzymatic lysis solutions was added to a 500 µl aliquot of cell suspension followed by incubation for 1 hour at 37°C. Then 600 mg of 0.1-mm-diameter zirconia/silica beads (BioSpec, Bartlesville, OK) were added to the lysate and the microbial cells were mechanically disrupted using a Mini-BeadBeater-96 (BioSpec, Bartlesville, OK) at 2100 rpm for 1 minute. Further isolation and purification of the total genomic DNA from lysates was done using QIAamp DNA mini kits (Qiagen, Valencia, CA). The isolated genomic DNA was in a final volume of 200 µl.

The DNA extractions were performed by three experimenters. Each of them finished three replicated DNA extractions per sample per DNA extraction method, resulting in 84 extractions per experimenter in total. These 84 extractions were randomly divided into five groups (16, 18, 16, 18 and 16 extractions) and extractions in each group were done on a given day. Furthermore, samples and enzymatic lysis solutions were prepared by a different investigator, so experimenters were blind to both the kinds of enzymatic lysis solutions used and the composition of samples. DNA concentrations were measured by using a Gemini XPS Fluorescence Microplate Reader (Molecular Devices, Sunnyvale, CA).
