# Supplementary material for: Evaluation of Methods for the Extraction and Purification of DNA from the Human Microbiome
Source: PLoS One. 2012 Mar 23;7(3):e33865. doi: 10.1371/journal.pone.0033865 (PMC3311548; doi:10.1371/journal.pone.0033865)
Supplement: Appendix S2 — A Poisson-Binomial mixture model to account for overdispersion in microbiome sampling. (DOCX) [file pone.0033865.s002.docx]

## Appendix S2

## A Poisson-Binomial mixture model to account for overdispersion in microbiome sampling

In testing representation our goal was to evaluate if the observed number of 16S rRNA reads per species, $\boldsymbol{j}$ (one of eleven), within a sample are similar to those expected while accounting for experimental variation between replicates due to 454 pyrosequencing. To do so we used a likelihood ratio test, accounting for overdispersion using a Poisson-Binomial mixture model. This likelihood ratio test assumes two models: a null simple model and an alternative more complex model. The null model assumes that the observed reads, $\boldsymbol{Y}_{\boldsymbol{ij}}$, per species $\boldsymbol{j}$ within replicate $\boldsymbol{i}$, regardless of the experimenter and the day, have an underlying multinomial distribution with parameters equal to the expected relative abundances, $\boldsymbol{p}_{\boldsymbol{1}}\boldsymbol{,}\boldsymbol{p}_{\boldsymbol{2}}\boldsymbol{,}\boldsymbol{\ldots}\boldsymbol{,}\boldsymbol{p}_{\boldsymbol{11}}$. To account for overdispersion we assumed that the total number of reads, $\boldsymbol{N}_{\boldsymbol{i}}$, per replicate $\boldsymbol{i}$ to be Poisson distributed with mean λ. The likelihood of the data given this simple model is,

$$P\left( \left\{ Y_{ij} \right\}, N_{i} | \left\{ p_{i} \right\}, \lambda\right)= P\left( \left\{ Y_{ij} \right\} | \left\{ p_{i} \right\}, N_{i} \right)P\left( N_{i} | \lambda\right)= \prod_{i} \frac{\lambda^{N_{i}}e^{\lambda}}{N_{i}!}\left( \frac{N_{i}!}{\prod_{j} Y_{ij}!} \right)\prod_{j} p_{j}^{Y_{ij}}$$

The likelihood of the alternative model is similar to the null except that it assumes that the replicates have a multinomial distribution different than what is expected and that the parameters of this model ${\hat{\boldsymbol{p}}}_{\boldsymbol{i}}$ are estimated based on the observed data and are equal to $\frac{\sum_{\boldsymbol{i}} \boldsymbol{Y}_{\boldsymbol{ij}}}{\sum_{\boldsymbol{i}} \boldsymbol{N}_{\boldsymbol{i}}}$. This alternative model still includes the Poisson model to account for sample variation. The likelihood ratio test we implemented compares the null and the alternative models using a strategy equivalent to that presented by Schütte et al. 2009 [61] and based on the bootstrap.
