# Supplementary figures and images for: Evaluation of Methods for the Extraction and Purification of DNA from the Human Microbiome
Source: PLoS One. 2012 Mar 23;7(3):e33865. doi: 10.1371/journal.pone.0033865 (PMC3311548; doi:10.1371/journal.pone.0033865)

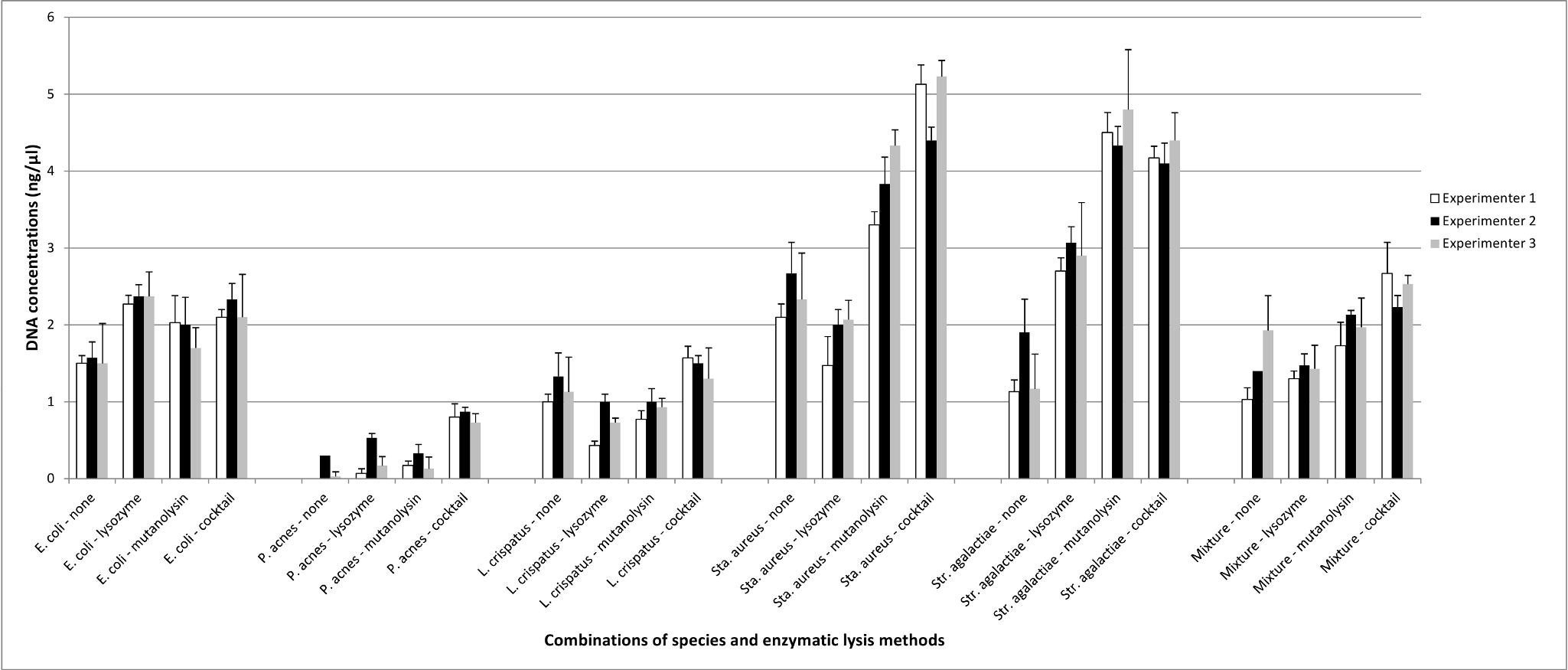

Supplement: Figure S1 — Combinations of species and enzymatic lysis methods. (TIF) [file pone.0033865.s003.tif]
